# Inhibition of Plasmodium Liver Infection by Ivermectin

**DOI:** 10.1128/AAC.02005-16

**Published:** 2017-01-24

**Authors:** António M. Mendes, Inês S. Albuquerque, Marta Machado, Joana Pissarra, Patrícia Meireles, Miguel Prudêncio

**Affiliations:** Instituto de Medicina Molecular, Faculdade de Medicina, Universidade de Lisboa, Lisbon, Portugal

**Keywords:** avermectins, ivermectin, Plasmodium, liver stage, malaria

## Abstract

Avermectins are powerful endectocides with an established potential to reduce the incidence of vector-borne diseases. Here, we show that several avermectins inhibit the hepatic stage of Plasmodium infection *in vitro*. Notably, ivermectin potently inhibits liver infection *in vivo* by impairing parasite development inside hepatocytes. This impairment has a clear impact on the ensuing blood stage parasitemia, reducing disease severity and enhancing host survival. Ivermectin has been proposed as a tool to control malaria transmission because of its effects on the mosquito vector. Our study extends the effect of ivermectin to the early stages of mammalian host infection and supports the inclusion of this multipurpose drug in malaria control strategies.

## INTRODUCTION

Despite recent achievements, malaria remains a formidable public health problem, primarily affecting children in the poorest regions of the world. Plasmodium parasites, the causative agents of malaria, are transmitted to humans as sporozoites (spz) through the bite of infected Anopheles mosquitoes. Injected spz travel to the liver and initiate an asymptomatic, yet obligatory, replication and differentiation phase (1). This intrahepatic developmental process culminates in the release of thousands of merozoites into the bloodstream, where they cyclically infect red blood cells, causing disease symptoms and originating gametocytes that warrant the progress of infection in the mosquito vector. Historically, successful malaria control interventions have combined effective vector control strategies, capable of interrupting transmission, and strong antiparasitic drugs that prevent disease and death. However, current tools are precarious and calls have recently been made for the development of new drug formulations or the repurposing of old drug formulations as valuable interventions to help control malaria infection (2).

Avermectins are a family of macrocyclic lactones that includes compounds presenting not only a best-in-class antiparasitic activity but also a strong insecticidal effect (3). Their impact on vector populations led to the suggestion of a potential role for avermectins in reducing the incidence of vector-borne disease (4). In particular, ivermectin, a semisynthetic member of the avermectin family, revolutionized the treatment of nematode and arthropod parasites in animals and is commonly used to treat neglected tropical diseases such as onchocerciasis, lymphatic filariasis, and strongyloidiasis (5–7). More recently, ivermectin has emerged as a potential tool for malaria control (4, 8–10), given its insecticidal effect (11–16), its ability to inhibit Plasmodium falciparum sporogony (17), its inhibitory effect on the blood stages of Plasmodium berghei (*in vivo*) and P. falciparum (*in vitro*) (18), and its ability to disrupt parasite transmission (19–21). However, no reports on the effect of avermectins on Plasmodium liver stages exist so far.

Here, we investigated the effects of avermectins on the liver stages of P. berghei parasites and showed that ivermectin is remarkably active against this stage of the malaria parasite's life cycle. These results provide further support for the potential of ivermectin, a drug that is already employed in mass drug administration (MDA) in regions where malaria is endemic and has an established impact on malaria transmission, as a multipurpose, multistage tool for malaria control.

## RESULTS

### Avermectins inhibit Plasmodium infection of human hepatoma cells *in vitro*.

**FIG 1:**
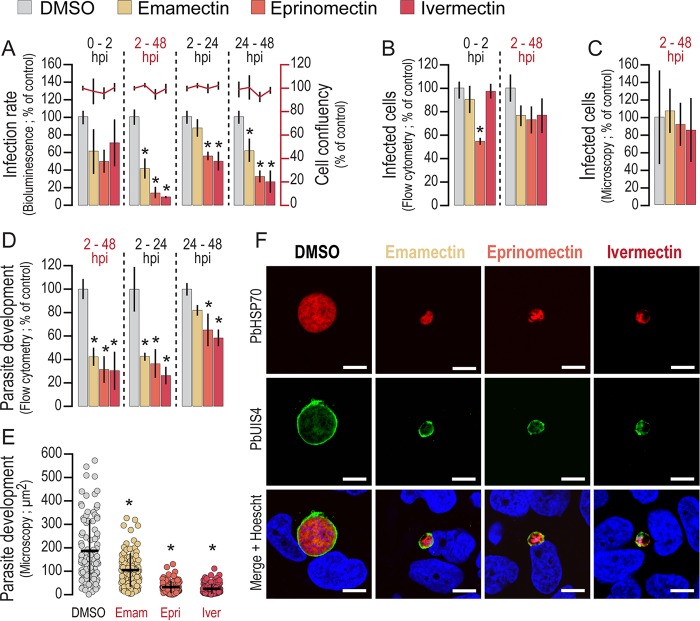
Avermectins inhibit the development of Plasmodium hepatic stages *in vitro*. (A) Huh7 cells were infected with luciferase-expressing P. berghei spz and treated with the IC_90_s of avermectins or with equivalent amounts of dimethyl sulfoxide (DMSO; control) for the times indicated. Total parasite loads (bioluminescence) and cell viability were assessed at 48 hpi. (B) Parasite invasion was quantified by flow cytometry at 2 hpi with GFP-expressing P. berghei spz by determining the percentage of GFP^+^ cells. (C) Hepatic parasite numbers at 48 hpi were quantified by immunofluorescence microcopy. (D, E) Parasite development at 48 hpi was assessed by flow cytometry (D) and immunofluorescence microscopy (E) by determining the fluorescence intensity of GFP^+^ cells and the area of developing parasites, respectively. (F) The effect of avermectin treatment on PVM structure, somatic integrity, and schizogony was analyzed by immunofluorescence microcopy employing antibodies against PbUIS4 (a PVM protein, in green), PbHSP70 (a heat shock protein that localizes to the parasite soma, in red), and the nuclear stain Hoechst (in blue). Representative confocal images show P. berghei hepatic forms treated with the IC_90_s of different avermectins or with dimethyl sulfoxide (control) from 2 to 48 hpi. Scale bars, 10 μm. Plots represent the mean values of at least three independent experiments with error bars indicating the standard deviations. The Mann-Whitney test was employed to assess the statistical significance of differences (*, *P* < 0.05).

The effect of emamectin, eprinomectin, and ivermectin on the *in vitro* infection of Huh7 cells by luciferase-expressing P. berghei parasites was measured by using a bioluminescence assay (22). Our data show that all three avermectins tested are active against Plasmodium liver stages, with 50% inhibitory concentrations (IC_50_s) of ∼2 μM, equivalent to 2.6, 2.2, and 2.1 μg/ml for emamectin, eprinomectin, and ivermectin, respectively (see Fig. S1 in the supplemental material). Treatment at different periods of infection further indicated that these avermectins act mainly during the parasite's intrahepatic development phase, as the strongest effect occurs when the drugs are added to the cells after completion of the invasion process (Fig. 1A). The invasion and development phenotypes in the presence of the compounds were further analyzed by a flow cytometry-based approach (23). These data suggested an effect of eprinomectin on the ability of the parasites to invade Huh7 cells, which was not observed with either emamectin or ivermectin (Fig. 1B). Furthermore, quantification of the infected cells at 48 h postinfection (hpi) by either flow cytometry (Fig. 1B) or immunofluorescence microscopy (Fig. 1C) showed no significant changes in the overall number of intracellular Plasmodium parasites. On the contrary, avermectins strongly impair the parasite's ability to develop inside cells. Measurement of green fluorescent protein (GFP) intensity in cells at 48 hpi, a correlate of parasite development, showed that avermectins inhibit parasite replication, particularly when present between 2 and 24 h of infection (Fig. 1D). This effect was confirmed by immunofluorescence microscopy analysis of infected cells incubated with drugs at their IC_90_s throughout the 48-h infection period (Fig. 1E and F). Nonetheless, treatment with avermectins does not disrupt parasitophorous vacuole membrane (PVM) formation and does not affect the localization of the PVM-resident protein, upregulated in spz 4, PbUIS4 (Fig. 1F) or of the circumsporozoite protein PbCSP (see Fig. S2). Avermectin treatment does significantly impact somatic integrity and schizogony, as the parasite's DNA appears to be strongly condensed (Fig. 1F; see Fig. S2). Overall, our results show that avermectins inhibit Plasmodium hepatic infection by impairing the parasite's ability to develop inside cells.

### Ivermectin decreases the liver Plasmodium load *in vivo*.

**FIG 2:**
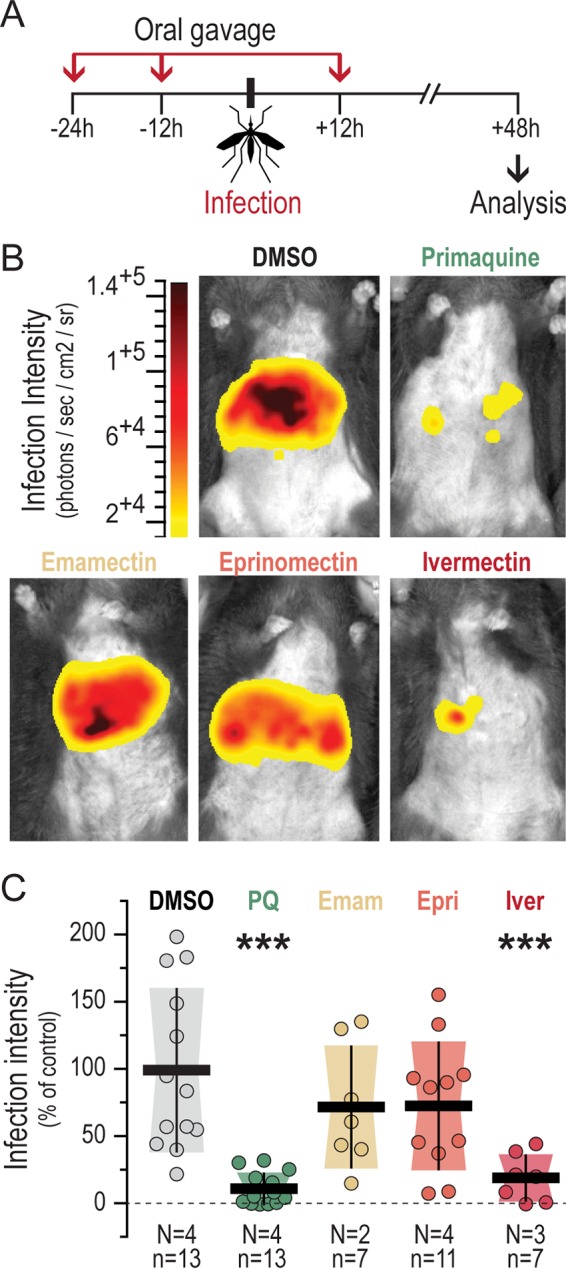
Ivermectin decreases liver infection *in vivo*. (A) Schematic illustration of the *in vivo* experimental setup. The arrows indicate when each avermectin was orally administered at 10 mg/kg. Vehicle- or primaquine (10 mg/kg)-treated mice were used as controls. P. berghei spz were delivered by the bites of 5 to 7 mosquitoes. (B) Representative bioluminescence images of mouse livers at 48 hpi. (C) Liver parasite infection loads in mice treated with the various compounds, relative to those in DMSO-treated controls. Dots represent the radiance intensity of individual liver areas relative to the average radiance of all DMSO-treated mice. The total number of mice (n) in each data set and the number of independent biological replicate experiments (N) performed are indicated. The statistical significance of differences from the DMSO-treated mice was calculated by employing the nonparametric Mann-Whitney test. *P* values are indicated above each data set (***, *P* < 0.001). Horizontal dark lines indicate the relative mean liver parasite infection load, whereas the vertical bars and shaded area represent the standard deviation of the mean. PQ, primaquine; Emam, emamectin; Epri, eprinomectin; Iver, ivermectin.

We further evaluated the effect of avermectins in the context of a physiologically relevant Plasmodium liver infection. Emamectin, eprinomectin, and ivermectin were administered to mice by oral gavage on a three-dose administration schedule of 10-mg/kg solutions in soybean oil (Fig. 2A). Mice were infected by mosquito bite injection of P. berghei spz 24 h after the first drug administration, and the parasite load was assessed 44 to 46 h after parasite administration. Our data show that ∼80% inhibition of liver infection *in vivo* is observed upon treatment with 10 mg/kg ivermectin but not upon treatment with emamectin or eprinomectin, an effect comparable to that obtained with an equivalent administration of primaquine, the only licensed liver stage antiplasmodial drug (24) (Fig. 2B and C). It should be noted that upon administration of 10 mg/kg ivermectin, some mice displayed behavioral signs of neurotoxicity. However, equally of note, strong impairment of infection was also observed when lower doses of ivermectin (1, 5, and 8 mg/kg) were administered following a similar regimen (see Fig. S3). Since avermectins have a strong insecticidal effect on Anopheles mosquitoes (8), we examined whether the observed effect on liver infection resulted from deficient mosquito-to-mammal parasite transmission because of an alteration of the mosquito feeding behavior on drug-treated mice. To investigate this, we initially inspected the mosquitoes' feeding pattern on drug- and vehicle-treated mice. Our data show that mosquito feeding behavior was not altered by the presence of avermectins in circulation (see Fig. S4A), whereas, not surprisingly, they strongly affected mosquito survival after feeding (see Fig. S4B). We then questioned whether the amount of Plasmodium spz deposited into the skin would be influenced by the circulating avermectins. The bioluminescence signal corresponding to the parasites deposited into mouse skin was measured immediately after a mosquito blood meal, revealing no significant differences between groups of mice (see Fig. S4C and D). Overall, our data show that although the presence of avermectins in circulation has no effect on the feeding behavior of Plasmodium-infected mosquitoes or on spz deposition into mammalian host skin, ivermectin displays a marked effect on P. berghei liver infection *in vivo*.

### Ivermectin impairs parasite development in the liver and impacts host survival.

**FIG 3:**
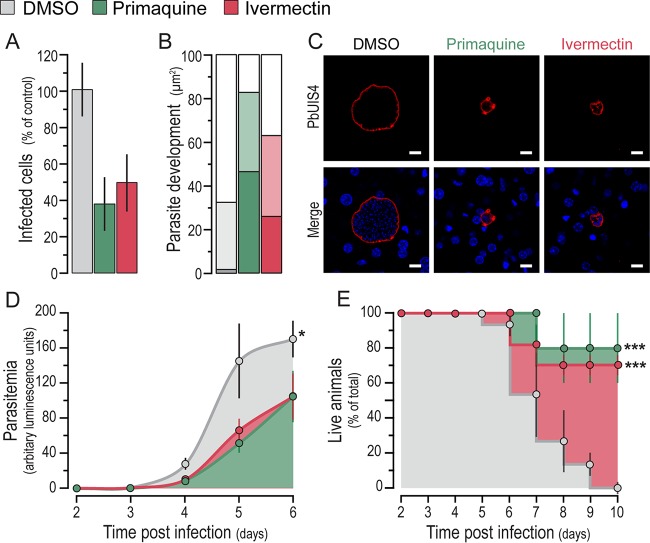
Ivermectin treatment inhibits parasite development in hepatocytes and improves the outcome of disease. (A) Parasite density per square millimeter of liver section following treatment with ivermectin, primaquine, or the vehicle at 48 hpi. (B) Effect of treatment on the parasite area as a correlate of parasite development at 48 hpi. Darker colors indicate parasite areas of <250 μm^2^, intermediate shading indicates areas between 250 and 750 μm^2^, and lighter bars indicate parasite areas of >750 μm^2^. (C) Representative confocal microscopy images of liver parasites in treated and control mice. Green, PbUIS4 labeling showing the PVM; blue, Hoechst nuclear staining. Scale bars,10 μm. (D) Assessment of the prepatency period and blood parasitemia by bioluminescence assays at various time points after spz administration and drug treatment as in Fig. 2A. The mean bioluminescence ± the standard errors of the pooled data of 15 mice from three biological replicate experiments is presented for each of the time points analyzed. A statistical analysis of the mean parasitemia observed across time was performed by using the nonparametric Friedman test, which indicated a significant difference, with a *P* value of <0.05 (*). (E) Mouse survival following spz administration and drug treatment as in Fig. 2A. The mean percentage of live animals ± the standard errors of the pooled data of 15 mice from three biological replicate experiments is shown for daily records, starting at the second day after spz administration and drug treatment and up to 10 days pi. The Mantel-Cox (log rank) test was employed to compare survival curves, indicating statistically significant differences, with a *P* value of <0.001 (***) for both the primaquine- and ivermectin-treated groups compared to the DMSO-treated controls.

To investigate the basis of the observed impairment of *in vivo* liver infection by ivermectin, infected mouse livers were analyzed by immunofluorescence microscopy at 46 hpi. Our analysis showed that ivermectin-treated mice had fewer (Fig. 3A) and significantly less developed (Fig. 3B and C) liver parasites than those of vehicle-treated control mice. We then investigated whether the ivermectin-dependent reduction of liver infection would impact the appearance of parasites in the blood and the ensuing pathology. To this end, infection was allowed to proceed past the liver stage and blood stage parasitemia, disease symptoms, and mouse survival were monitored for 10 days following parasite administration. Our results show that the impairment of liver parasite development caused by ivermectin treatment affects the onset of blood parasitemia (Fig. 3D) and, most importantly, has a clear impact on host survival, with 80% of the treated mice surviving for 10 days, compared with all of the control mice dying with symptoms of experimental cerebral malaria (ECM) within the same period (Fig. 3E). Overall, these results show that ivermectin significantly abrogates liver infection by impairing parasite development and survival in hepatic cells, which impacts the ensuing blood parasite burden and contributes to enhanced host survival.

## DISCUSSION

Avermectins are widely used for protection against a wide spectrum of parasitic diseases. Various avermectins have been synthesized, with different antiparasitic efficacies and pharmacokinetic profiles, including one, ivermectin, that is safe and well tolerated in humans. Several studies have shown that ivermectin has a strong insecticidal effect that could be explored as a potential tool against malaria transmission (9). Besides, inhibitory effects of ivermectin against the sporogonic and blood stages of the Plasmodium life cycle have been described, highlighting its potential as a multistage malaria intervention strategy (17, 18).

The effect of avermectins on the liver stage of Plasmodium infection had hitherto not been assessed. Nevertheless, the relatively low numbers of parasites that infect hepatocytes, coupled with the obligatory and asymptomatic natures of this phase of infection, make the liver a privileged target for prophylactic intervention (1, 25). Moreover, the existence of Plasmodium species capable of forming hypnozoites, parasite forms that remain dormant in the liver for long periods of time, demands effective ways of clearing hepatic parasites before they can cause disease relapses. We originally described a potential inhibition of Plasmodium hepatic infection by ivermectin as part of the results obtained in a drug screen targeting Plasmodium liver stages (26). We now demonstrate that ivermectin is the only compound of the avermectin family assessed in the present study that is active against Plasmodium liver stages *in vivo*. We confirm that ivermectin treatment strongly decreases parasite load *in vivo*, to a degree that is comparable to that of primaquine. Ivermectin strongly inhibits parasite development in the liver, which has a strong impact on the ensuing pathology and host survival, decreasing the magnitude of the blood stage infection and protecting from cerebral disease.

The exact mechanism of action of primaquine remains elusive but may involve impairment of the parasites' mitochondrial metabolism or the production of highly reactive metabolites that generate intracellular oxidative potentials (reviewed in references 24 and 27). Azithromycin, another drug with demonstrated activity against P. berghei liver stages, is proposed to act by blocking apicoplast development, leading to impaired parasite maturation (28). However, the molecular mechanism of ivermectin inhibition of Plasmodium liver stages is currently unknown. In the mosquito, the primary target of ivermectin is the invertebrate glutamate-gated chloride channel (GluCl) (29). Ivermectin has also been shown to kill the parasite's blood stages by blocking the nuclear import of the P. falciparum signal recognition particle (SRP), a family of six polypeptides involved in protein targeting to the endoplasmic reticulum (18). Recently, ivermectin was shown to be a ligand for farnesoid X receptor (NR1H4) (30), whose activation has a critical role in regulating the homeostasis of glucose, a molecule that plays a pivotal role during hepatic Plasmodium infection (31).

A recent modeling study has shown that adding ivermectin to mass treatment strategies with artemether-lumefantrine can help reduce/interrupt malaria transmission (32), and efforts are being made to develop a suitable slow-release formulation (33). Our results now show that besides its recognized effect on mosquitoes, ivermectin inhibits Plasmodium development inside hepatic cells, the obligatory initial stage of the malaria parasite's infection of its mammalian host. Thus, our study lends further support to the use of ivermectin as a tool for malaria control and warrants further investigation of the impact of ivermectin MDA on this devastating disease.

## MATERIALS AND METHODS

### Mice, parasites, and reagents.

Male C57BL/6 mice 6 to 8 weeks old were purchased from Charles River and housed in facilities of the Instituto de Medicina Molecular (iMM), Lisbon. Experimental procedures were performed in accordance with the Federation of European Laboratory Animal Science Associations guidelines and iMM regulations. In experiments involving blood stage infections, mice were euthanized at the first behavioral signs of onset of ECM, and this was considered the experimental endpoint. When no ECM was observed, mice were euthanized at day 10 postinfection (pi), the end of the time frame for the development of this phenotype. A GFP/luciferase-expressing P. berghei ANKA transgenic parasite line (676m1cl1) was used in all experiments. Mosquitoes were bred at the insectary facility of the iMM. All of the chemicals used were obtained from Sigma-Aldrich (St. Louis, MO, USA).

### Quantification of *in vitro* parasite infection.

For IC_50_ and IC_90_ determinations, compounds were added to the cells prior to spz addition and incubated for 48 h. In all other experiments, compounds were used at their IC_90_s and incubated for the times indicated. The infection load of human hepatoma Huh7 cells was determined by bioluminescence measurements as previously described (22). Invasion and intracellular parasite development were assessed by determining the percentage of GFP^+^ cells at 2 hpi and GFP intensity at 48 hpi, respectively, as previously described (23). The Mann-Whitney test was employed to assess the statistical significance of differences (*P* < 0.05; GraphPad Prism v5).

### Immunofluorescence microscopy analysis of *in vitro* and *in vivo* hepatic infections.

For *in vitro* studies, cells seeded onto coverslips were fixed at 48 hpi with 4% (vol/vol) paraformaldehyde for 10 min at room temperature (RT), permeabilized/blocked with 0.1% (vol/vol) Triton X-100–1% (wt/vol) bovine serum albumin in 1× phosphate-buffered saline (PBS) for 30 min at RT, and incubated with anti-Hsp70 and anti-UIS4 antibodies for 1 h at RT, followed by adequate secondary antibodies and Hoechst 33342 for 1 h at RT. For *in vivo* studies, fixed sections of livers collected at 48 hpi (intravenous [i.v.] administration of 30,000 spz) were similarly stained. Images were acquired with a Zeiss 510 Meta confocal microscope and a Zeiss Axiovert 200 M microscope and then processed with the ImageJ software.

### Quantification of *in vivo* hepatic infection and blood stage parasitemia.

*In vivo* parasite loads in mice were determined by real-time *in vivo* imaging 30 min and 44 to 46 h after 15 min of exposure to five to seven infected Anopheles stephensi mosquitoes with the *in vivo* IVIS Lumina Imaging System as previously described (22). Additionally, real-time PCR analysis of specific P. berghei 18S rRNA and mouse hypoxanthine guanine phosphoribosyl transferase (HPRT)-encoding housekeeping genes in infected livers collected at 44 to 46 hpi was performed as previously described (34). The nonparametric Mann-Whitney test was applied to assess the statistical significance of differences between liver parasite loads (*P* < 0.001; GraphPad Prism v5). Blood stage parasitemia following the i.v. injection of 5,000 spz was assessed by collecting 5 μl of tail blood between days 2 and 6 pi in 50 μl of lysis buffer. Bioluminescence was then measured following the addition of 50 μl of d-luciferin dissolved in firefly luciferase assay buffer to 30 μl of lysate with an Infinite M200 multiplate reader (Tecan). The nonparametric Friedman test was applied to assess the statistical significance of differences between the mean parasitemias observed across time in mice subjected to the different drug treatments (*P* < 0.05; GraphPad Prism v5).

## Supplementary Material

Supplemental material
