# Supplementary material for: Inhibition of Plasmodium Liver Infection by Ivermectin
Source: Antimicrob Agents Chemother. 2017 Jan 24;61(2):e02005-16. doi: 10.1128/AAC.02005-16 (PMC5278742; doi:10.1128/AAC.02005-16)
Supplement: Supplemental material [file supp_61_2_e02005-16__index.html]

Inhibition of Plasmodium Liver Infection by Ivermectin — Supplemental material 

# Inhibition of Plasmodium Liver Infection by Ivermectin

## Supplemental material

- Supplemental file 1 -

  Figures S1 to S4

  PDF, 823K
