# Supplementary material for: Inhibition of Plasmodium Liver Infection by Ivermectin
Source: Antimicrob Agents Chemother. 2017 Jan 24;61(2):e02005-16. doi: 10.1128/AAC.02005-16 (PMC5278742; doi:10.1128/AAC.02005-16)
Supplement: Supplemental material [file AAC.02005-16_zac002175904s1.pdf]

Inhibition of *Plasmodium* liver infection by ivermectin

António M. Mendes, Inês S. Albuquerque, Marta Machado, Joana Pissarra, Patrícia Meireles, Miguel Prudêncio

Supplemental Material (4 pages)

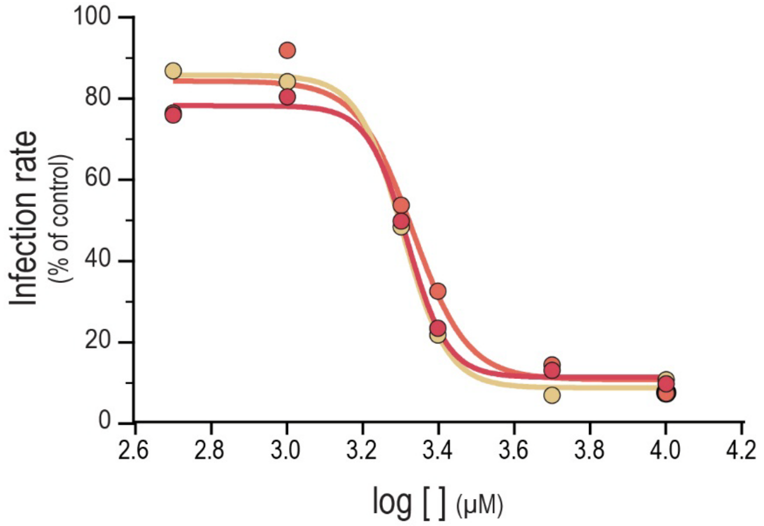

Emamectin	IC50=2.6μM / 2621.47 ng/ml	IC90=2.7μM / 2722.29 ng/ml
Eprinomectin	IC50=2.2μM / 1980.22 ng/ml	IC90=3.1μM / 2790.31 ng/ml
Ivermectin	IC50=2.1μM / 1837.71 ng/ml	IC90=2.7μM / 2362.77 ng/ml

FIG. S1. Avermectins inhibit *Plasmodium* hepatic infection *in vitro*. Dose-dependent effect of various avermectins on *P. berghei* infection of Huh7 hepatoma cells, as measured by bioluminescence. Compounds were added to the cells prior to infection with *P. berghei* sporozoites and maintained in the culture medium for 48 hours. Data represent the mean of three independent experiments.

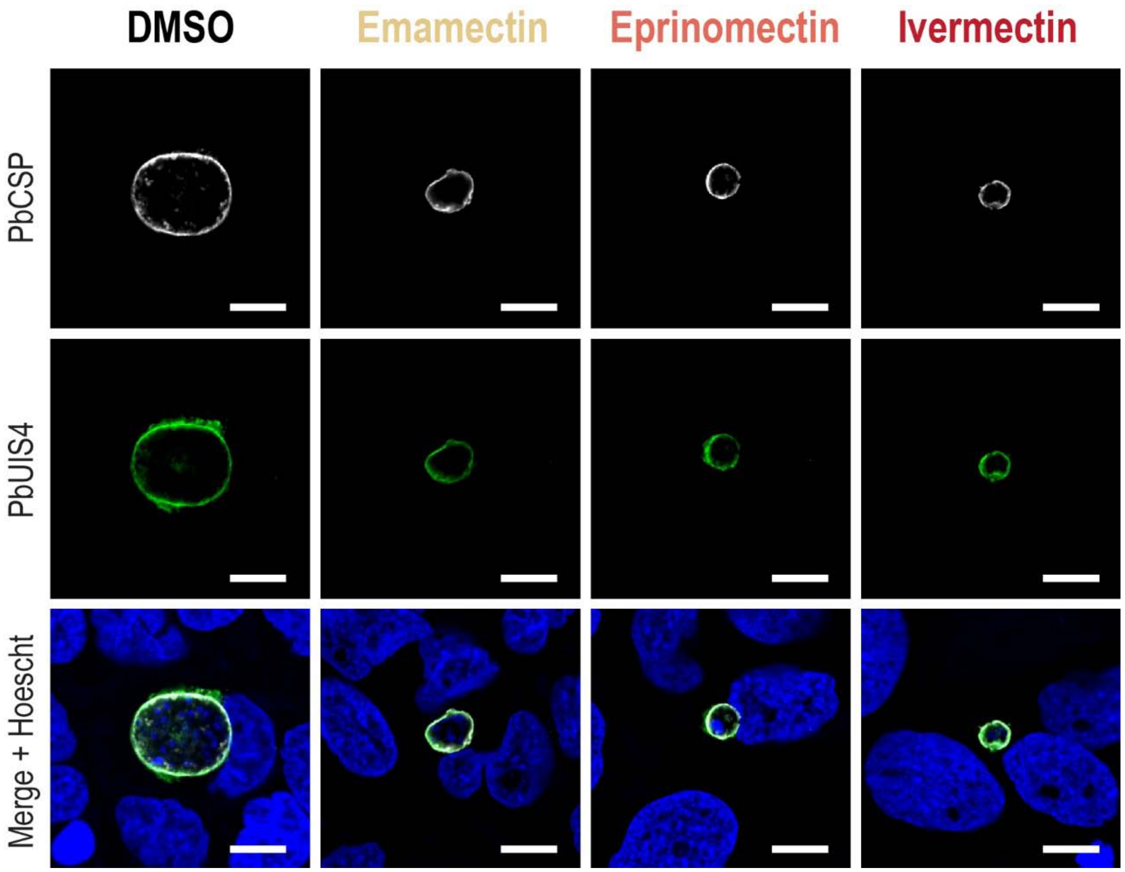

FIG. S2. Avermectins do not disrupt the localization of PbCSP at the parasite surface. Representative confocal immunofluorescence microscopy images of *P. berghei* hepatic forms, following treatment with IC90 concentrations of different avermectins or DMSO control, from 2 to 48 hpi. White: PbCSP; Green: PbUIS4; Blue: Hoechst nuclear stain (blue). Scale bar, 10 µm.

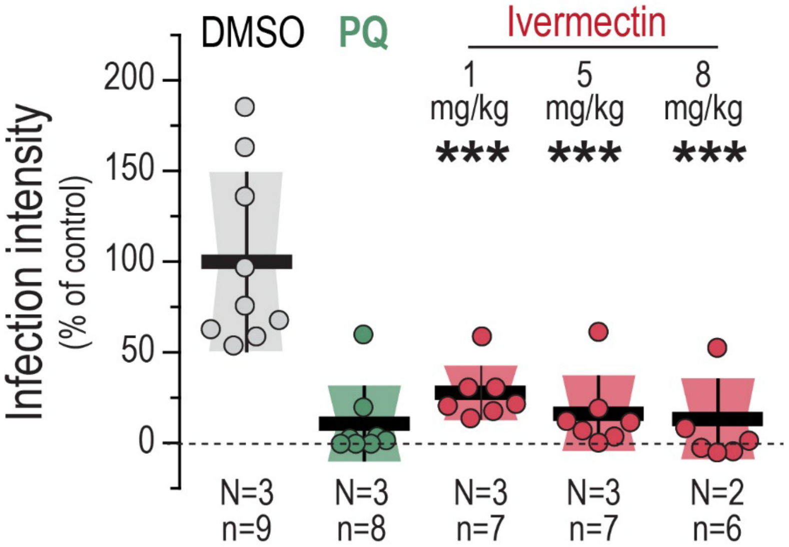

FIG. S3. Liver parasite loads of mice treated with different dosages of ivermectin. Dots indicate the normalized expression of the *P. berghei* 18S gene to the mouse HPRT as determined by qRT-PCR in total liver RNA and as percentage of DMSO treated controls. Primaquine (PQ)-treated mice (10mg/kg) were used as a positive control. The total number of mice (n) in each dataset as well the number of independent biological replicate experiments (N) performed are indicated. Statistically significant differences relative to DMSO-treated mice were calculated by employing the non-parametric Mann-Whitney test and the P-values are indicated above each dataset (*** P<0.001). Horizontal dark lines indicate the relative mean liver parasite infection load whereas the vertical bars and shaded area represent the standard deviation of the mean.

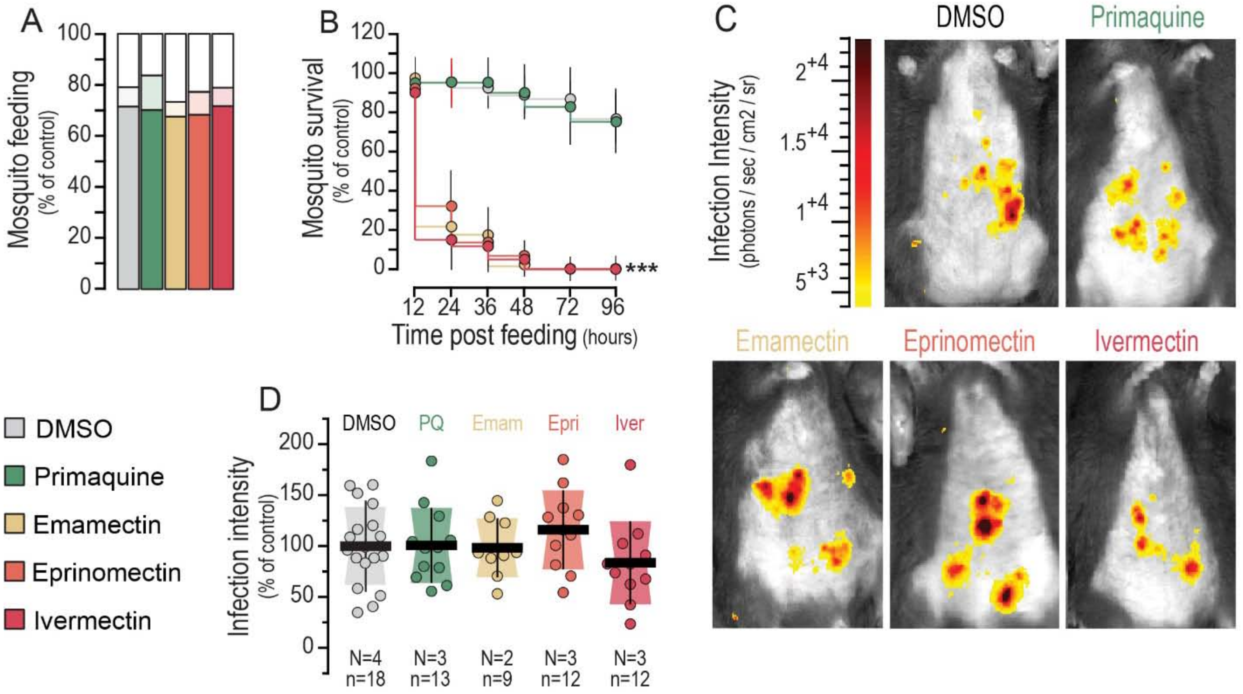

FIG. S4. Treatment with avermectins does not affect the feeding behavior or the capacity of mosquitoes to deliver sporozoites. A) Numbers of infected mosquitoes that ingested a complete blood-meal (darker color), a partial blood-meal (lighter color) or that did not ingest a blood-meal (white) following exposure to mice treated with various avermectins or to DMSO- and primaquine-treated control mice. **B)** Mosquito survival at various time points after ingestion of a blood meal on treated and control animals. The mean percentage of live mosquitoes ± standard errors of the pooled data from at least 3 biological replicate experiments is shown for every 12 hours up to 96 hours post feeding on drug treated mice. The Mantel-Cox (logRank) test was employed to compare survival curves indicating statistically significant differences, with a P value <0.001*** for the survival of mosquitoes fed on mice treated with either Ivermectin, Eprinomectin or Emamectin but not for mosquitoes fed on mice treated with Primaquine. **C** and **D)** In vivo bioluminescence imaging of the overall number of sporozoites deposited by a group of 5 to 7 infected mosquitoes on the skin of treated and control mice, assessed 30 minutes post exposure to mosquitoes. **C)** Representative images of sporozoite loads in the skin of treated mice. **D)** Rradiance intensity of the exposed area of each mouse relative to the average radiance obtained for all DMSO-treated mice. The dark line indicates the relative mean infection load, and the shaded colored area represents the standard deviation of the mean.
